# Grafting and Early Expression of Growth Factors from Adipose-Derived Stem Cells Transplanted into the Cochlea, in a Guinea Pig Model of Acoustic Trauma

**DOI:** 10.3389/fncel.2014.00334

**Published:** 2014-10-20

**Authors:** Anna Rita Fetoni, Wanda Lattanzi, Sara Letizia Maria Eramo, Marta Barba, Fabiola Paciello, Chiara Moriconi, Rolando Rolesi, Fabrizio Michetti, Diana Troiani, Gaetano Paludetti

**Affiliations:** ^1^Department of Head and Neck Surgery, Università Cattolica del Sacro Cuore, Rome, Italy; ^2^Institute of Anatomy and Cell Biology, Università Cattolica del Sacro Cuore, Rome, Italy; ^3^Latium Musculoskeletal Tissue Bank, Rome, Italy; ^4^Institute of Physiology, Università Cattolica del Sacro Cuore, Rome, Italy

**Keywords:** adipose-derived stem cells, cochlea, noise-induced hearing loss, gene expression, *in vivo* migration

## Abstract

Noise exposure causes damage of multiple cochlear cell types producing permanent hearing loss with important social consequences. In mammals, no regeneration of either damaged hair cells or auditory neurons has been observed and no successful treatment is available to achieve a functional recovery. Loads of evidence indicate adipose-derived stem cells (ASCs) as promising tools in diversified regenerative medicine applications, due to the high degree of plasticity and trophic features. This study was aimed at identifying the path of *in vivo* cell migration and expression of trophic growth factors, upon ASCs transplantation into the cochlea, following noise-induced injury. ASCs were isolated in primary culture from the adipose tissue of a guinea pig, transduced using a viral vector to express the green fluorescent protein, and implanted into the *scala tympani* of deafened animals. Auditory function was assessed 3 and 7 days after surgery. The expression of trophic growth factors was comparatively analyzed using real-time PCR in control and noise-injured cochlear tissues. Immunofluorescence was used to assess the *in vivo* localization and expression of trophic growth factors in ASCs and cochleae, 3 and 7 days following homologous implantation. ASC implantation did not modify auditory function. ASCs migrated from the perilymphatic to the endolymphatic compartment, during the analyzed time course. Upon noise exposure, the expression of chemokine ligands and receptors related to the PDGF, VEGF, and TGFbeta pathways, increased in the cochlear tissues, possibly guiding *in vivo* cell migration. Immunofluorescence confirmed the increased expression, which appeared to be further strengthened by ASCs’ implantation. These results indicated that ASCs are able to migrate at the site of tissue damage and express trophic factors, upon intracochlear implantation, providing an original proof of principle, which could pave the way for further developments of ASC-based treatments of deafness.

## Introduction

Sensorineural hearing loss (SNHL) is one of the most common disabilities affecting adults and pediatric patients. It has been estimated that about 300 million adults and 32 million children suffer from deafness with relevant consequences on the communicative abilities and cognitive development particularly in children (Paludetti et al., 2012; Géléoc and Holt, 2014). Noise-induced hearing loss (NIHL) is the second most common sensorineural hearing deficit, after age-related hearing loss (presbyacusis), and the leading cause of preventable SNHL in the industrialized world (Fetoni et al., 2011). NIHL affects approximately 22 million Americans (http://www.nidcd.nih.gov). In Europe, 7% of workers are affected by hearing impairment and the cost of NIHL represents about 10% of total compensation costs for occupational diseases (https://osha.europa.eu). The demographic aspects of SNHL along with the inadequacy of conventional treatments, suggest the priority and the challenge for research to decrease the effects of deafness introducing innovative therapeutic approaches. In most cases, the cause for SNHL is directly or indirectly linked to oxidative stress-induced degeneration and death of hair cells (Henderson et al., 2006; Fetoni et al., 2010; Maulucci et al., 2014), along with loss of supporting cells and spiral ganglion neurons (Wang et al., 2002; Zilberstein et al., 2012). Among several different cochlear damaging mechanisms, processes leading to NIHL are well defined, thus this condition may be used as a model for SNHL (Fetoni et al., 2008). Noise stresses the cochlea metabolically and mechanically at several levels, leading to different forms of damage. In hair cells, noise can lead to overdriving of the mitochondria, excitotoxicity at the junctions between the inner cells and afferent auditory nerve fibers, and ischemia/reperfusion effects. These can lead to the increase of reactive oxygen species, resulting in DNA and cell membrane damages, and acting as a putative trigger for apoptotic cell death (Henderson et al., 2006). In mammals, unlike avian and non-mammalian vertebrates, no regeneration of either damaged hair cells or auditory neurons has been observed following SNHL (Bermingham-McDonogh and Reh, 2011; Ronaghi et al., 2012). At present, the available treatments for patients suffering from severe SNHL are exclusively based on sound amplification (hearing aids) and/or cochlear implants. However, only one out of five people who could benefit from a hearing aid actually wears one. Cochlear implants currently represent the gold standard therapy for severe to profound SNHL, despite their limited success in achieving hearing improvements. This is true in particular in children, where the use of cochlear implants does not restore normal hearing, due to poor innervation, which limits the perspective performance of an implant. Promising lines of research have focused on regenerative strategies based on stem cell transplantation to repair the damaged cochlear tissues by either replacing damaged cells or secreting factors that enhance the survival and/or proliferation of endogenous cells (Bernardo et al., 2009; da Silva Meirelles et al., 2009; Lai et al., 2010; Chen et al., 2012). A fully functional hearing relies on the precisely ordered architecture of the mammalian organ of Corti, characterized by several functionally specialized cell types, with a high degree of integration, along with synapses between the neuroepithelium and the spiral ganglion neurons (Brigande and Heller, 2009; Ronaghi et al., 2012). Therefore, the chance to achieve effective regeneration of the diversified cell types within the organ of Corti (hair cells, supporting cells, and spiral ganglion neurons) is strongly hampered by several technical and conceptual issues.

Regenerative medicine applications are dramatically increasing their range of applications, in both experimental field and in selected clinical settings. This is mainly due to the establishment of adipose tissue as a valuable source of somatic stem cells, due to its abundance and ease of retrieval, the rapid cell isolation and culture expansion procedures, and the inherent biological properties (Barba et al., 2013). Adipose-derived stem cells (ASCs) are multipotent stem cells residing in the stromal vascular fraction of fat, displaying extensive plasticity and multilineage differentiation potential (Bourin et al., 2013). ASCs are indeed able to differentiate toward all mesodermal lineages, including adipose tissue and other specialized connective tissues (i.e., bone, cartilage, and muscles) (Zuk et al., 2001; Bunnell et al., 2008; Saulnier et al., 2011). Despite this high plasticity, the claim that mesenchymal stem cells (MSCs) could be capable of trans-differentiation along non-mesodermal lineages is strongly debated. In particular, the question of a possible neural trans-differentiation of MSCs, such as ASCs, is still debated and controversial (Hu et al., 2013; Heng et al., 2014; Yang et al., 2014). Nonetheless, converging evidence has indicated that ASCs express and secrete an array of growth factors, including neurotrophic factors, which may explain their documented trophism and sustain the regeneration of both mesodermal and non-mesodermal organs and tissues (Saulnier et al., 2009; Lattanzi et al., 2011; Liu et al., 2013).

Based on this background, in this study, we have tried to lay the foundation for a possible ASC-based regenerative approach in the noise-injured cochlea, with the aim of clarifying the first issues that should arise when a cell-based treatment is developed: where are cells supposed to migrate and localize upon *in vivo* inoculation? Which signals are attracting cells to migrate toward a specific site? Could the inoculation procedure be detrimental, given the delicate and narrow anatomical location? Do cells express trophic factors *in vivo* to counteract the ischemic insult and/or support cell repair mechanism?

We have thus investigated the expression of biologically relevant growth factors and chemokines, known to be involved in MSC migration, angiogenic features, and overall tissue trophism.

## Materials and Methods

### Animals

**Figure 1:**
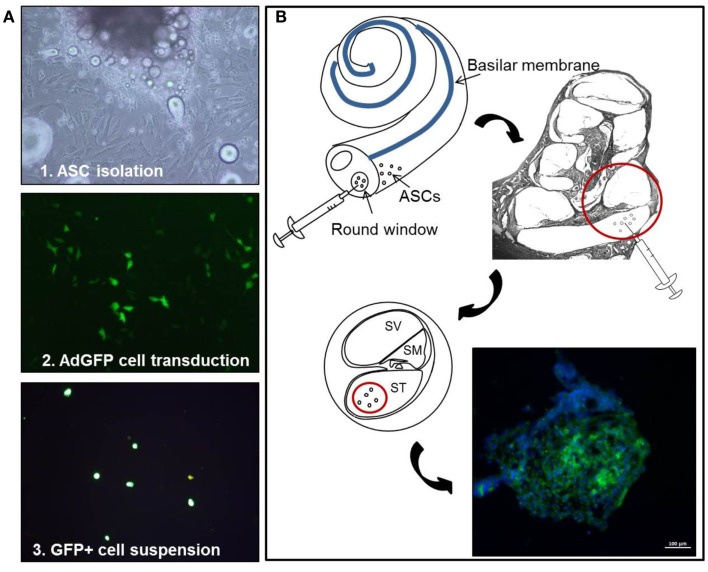
**Experimental design**. Schematic representation of the cell-based experimental procedure, showing **(A)** ASC primary culture and adenoviral mediated transduction, and **(B)** implantation via the round window: GFP-positive cells, with DAPI-stained nuclei, form multicellular aggregates. In this and following figures, ASCs, adipose stem cells; GFP, green fluorescent protein; DAPI, 4,6diammino-2-phenylindole.

A total of 40 adult Hartley guinea pigs (age 3 months, weight 250–350 g) with normal Preyer’s reflex were used in this study. The animals were randomized into the following groups: 8 animals were used as control group (Ctrl) with normal hearing in all the experimental procedures; the same animals underwent lipectomy for the isolation of autologous ASCs; 8 animals were used as noise control group (Noise) and were deafened with our noise exposure paradigm; 12 animals without prior noise deafening underwent green fluorescent ACS transplantation in the right ear (Sham + ASCs,) and vehicle injection in the left ear (Sham + vehicle); and finally, 12 animals were deafened and then (24 h afterward) implanted in the right ear with green fluorescent ASCs (Noise + ASCs) and in the left ear with vehicle (Noise + vehicle). For each animal in the unexposed and noise-exposed experimental groups, the ASC suspension was inoculated into the right ear, while a sham injection of culture medium was performed in the contralateral ear of each, as a matched internal control to increase reproducibility and cope with inter-individual variability and decrease the amount of animals used *in vivo* experiments. Throughout the experiment, animals were housed two per cage with temperature (22–23°C) and humidity controlled (60 ± 5%), under a 12 h light/dark cycle with food (4RF21; Mucedola, Milan, Italy) and water available *ad libitum*. All efforts were made to minimize animal suffering and to reduce the number needed for the experiments, in accordance with the European Community Council Directive, dating November 24, 1986 (86/609/EEC). All procedures were performed in compliance with the Laboratory of Animal Care and Use Committee of the *Università Cattolica del Sacro Cuore*, School of Medicine (Rome, Italy) and were approved by the Italian Department of Health. The entire experimental design is shown in Figure 1.

### Deafening procedure

Twenty animals (Noise = 8, Noise + ASCs = 12) were deafened before implantation as described in previous papers (Fetoni et al., 2008, 2010). The acoustic trauma was induced by a continuous pure tone generated by a waveform generator (LAG-120B, Audio Generator, Leader Electronics Corporation, Yokohama, Japan) and amplified by an audio amplifier (A-307R, Pioneer Electronics, Long Beach, CA, USA). The animals were deeply anesthetized (ketamine hydrochloride 25 mg/kg, xylazine 5 mg/kg, and acepromazine maleate 1.5 mg/kg body weight), placed in a soundproof room and exposed for 60 min, 5 days consecutively, to a 120 dB SPL pure tone sound at a frequency of 6 kHz. The sound was generated by a waveform generator (LAG-120B), amplified by an audio amplifier (A-307R), and symmetrically presented in open field by a dome tweeter (TW340X0, Audax, Chateau du Loir, France) positioned 10 cm in front of the animal’s head. Sound level was measured using a calibrated 1/4″ microphone (Model 7017, ACO Pacific Inc., Belmont, CA, USA) and a calibrated preamplifier (Acoustic Interface System, ACO Pacific Inc.).

### Cell culture and genetic engineering

All reagents used for cell culture were purchased from Euroclone (Milan, Italy), unless otherwise specified. MSCs were isolated in primary culture from pooled visceral adipose tissue specimens collected from both the neck and the abdomen regions of control animals (Ctrl), according to standardized procedures, already described elsewhere (Parrilla et al., 2011). Briefly, upon extensive washing in PBS and digestion with 0.1% collagenase (Lonza, Basel, Switzerland), the tissue lysate was filtered through a 100-μm mesh filter and centrifuged. The cell pellet was seeded into a T75 tissue culture flask using Dulbecco’s modified Eagle’s Medium (DMEM) with 4.5 g/L glucose, 0.1% l-glutamine, 0.1% penicillin/streptomycin solution, and 10% fetal bovine serum. Once isolated in primary culture, adherent cells were sub-cultured up to 3–4 culture passages. The “mesenchymal” immunophenotype was confirmed by flow cytometry, as previously described (Saulnier et al., 2009).

Subconfluent ASCs were then transduced using a defective adenoviral vector, carrying the green fluorescent protein (Ad-eGFP) as a reporter gene, as previously described (Pola et al., 2004; Parrilla et al., 2010). After 24 h, the number of green fluorescent cells was counted under fluorescence microscopy (Figure 1A), yielding a 80% transduction efficiency (Lattanzi et al., 2011). For the animal treatments, cells were harvested, washed twice, and suspended in PBS at a final density of 1.5 × 10^5^ cells/μl. To ensure that cell washing was sufficient to remove any possible residual free Ad-eGFP particle, an aliquot of the cell suspension was co-cultured with non-transduced ASCs up to 72 h. No GFP-positive cell was observed in non-transduced ASCs up to the latest tested time point (see Supplemental Material).

### Surgical procedure and ASC implantation via the round window

Surgery was performed on 12 Sham + ASCs and 12 Noise + ASCs animals. The guinea pigs were deeply anesthetized to undergo inoculation of green fluorescent ASCs (right ear), and sham treatment (left ear). The animals were placed on a heating pad to maintain body temperature at 37–38°C. The head and neck were shaved with 70% ethanol and a retroauricular incision was made under sterile conditions. Local anesthetic (2% lidocaine) was also delivered subcutaneously to the site of incision. The overlying muscles were removed in order to expose the *bulla tympani*, which was opened with a 0.8-mm diamond drilling burr. Using a stereomicroscope (Carl Zeiss OPMI^®^ pico; Carl Zeiss, Goettingen, Germany), the promontory and the round window membrane of the cochlea were identified. A 20-μL microsyringe (Hamilton microsyringe, Bonaduz, GR, Switzerland) was prefilled with green fluorescent ASC suspension (1.5 × 10^5^ cells/μl), 5 μL were spilled out, then (Figure 1B) the needle was inserted into the round window and 10 μl of perfusate were injected slowly at the speed of 1 μL/min into the *scala tympani* at the basal cochlear turn of the right cochleae (Nishimura et al., 2009; Pandit et al., 2011; Chen et al., 2012). The needle of the microsyringe was kept in the inner ear for several minutes to prevent the liquid flowout. A small piece of fascia was placed on the round window. After injection, the hole was plugged with dental cement in order to prevent any secondary otitis media, and the incision was closed with sutures. After the surgical procedure in the right ear, the same procedure was performed to access the left cochlea and inject the vehicle (10 μl PBS). All animals received anti-infectious prophylaxis using 100 mg/kg cefazolin, administered subcutaneously at the time of surgery, and their overall conditions were constantly monitored.

### Auditory function evaluation (Auditory brainstem responses, ABR)

Hearing function was evaluated in all animals by measuring ABR at low (2, 4 kHz), intermediate (6, 8, 12 kHz), and high (16, 20 kHz) frequencies, before and after the end of noise exposure, and at day 3 and day 7 after the surgery. Animals were mildly anesthetized with half dose of the anesthetic cocktail and placed in a soundproof room. Three stainless steel recording electrodes were subcutaneously inserted posterior to the tested pinna (active), vertex (reference), and contralateral pinna (ground). A computer-controlled TDT System 3 (Tucker–Davis Technologies, Alachua, FL, USA) data acquisition system with real-time digital signal processing was used to record the ABR and generate the auditory stimulus. Tone bursts ranging from 2 to 20 kHz (rise/fall time, 2 ms; total duration, 2 ms; repetition rate, 21/s) were presented monaurally in an open field using a horn tweeter (Tucker–Davis Technologies). Responses were filtered (100–3000 Hz band-pass), digitized and averaged across 1000 discrete samples at each frequency-level combination. Thresholds were determined by increasing the intensity of the tone in 5 dB steps starting at 0 dB and increasing to 100 dB or until the ABR response was detected. Then, the stimulus intensity was decreased in 5 dB steps until the latency-appropriate response disappeared. The threshold value was defined as the lowest intensity able to evoke an appropriate ABR response (Fetoni et al., 2010, 2013, 2014).

### Gene expression analysis in cochlear tissues and in ASCs

In order to define the molecular mechanisms possibly involved in the *in vivo* migration and “homing” of ASCs into the cochlea, we have analyzed the expression of genes encoding selected growth factors and corresponding receptors in both cochlear tissues and ASCs *ex vivo* (i.e., prior to *in vivo* transplantation), using reverse-transcription PCR (rtPCR) and real-time quantitative PCR (qPCR).

In particular, tissue expression analysis was performed comparatively in the following microdissected portions of the cochlea, in both noise-exposed (*n* = 3) and control unexposed animals (*n* = 3): organ of Corti, spiral ganglion, and *stria vascularis*. The tissue samples were homogenized using Trizol protocol (Invitrogen, Carlsbad, CA, USA), followed by RNA cleanup using the RNeasy MinElute Cleanup Kit (Qiagen, Hilden, Germany), as previously described (Bernardini et al., 2010). Total RNA served as template rtPCR and the cDNA was amplified using qPCR, carried out as described elsewhere (Lattanzi et al., 2013). The following genes were analyzed as targets: chemokine (C-C motif) ligand 2 (Ccl2), chemokine (C-C motif) ligand 5 (Ccl5/Rantes), transforming growth factor β (Tgfβ), vascular endothelial growth factor A (Vegf-A), Vegf-receptors 1-2-3 (Vegfr1, Vegfr2, Vegfr3), platelet-derived growth factor, α (PdgfA), Pdgf-receptor (Pdgfr). The 2^-ΔΔ^*^Ct^* method was applied to calculate relative quantity (RQ) in gene expression using the housekeeping gene, encoding the glyceraldehyde 3-phosphate dehydrogenase (Gapdh), for data normalization (Lattanzi et al., 2007).

Gene expression in ASCs was analyzed *in vitro* (i.e., prior to *in vivo* inoculation), using two-step rtPCR. To this aim, total RNA was isolated from confluent cells using the RNeasy mini kit (Qiagen, Hilden, Germany), as already described elsewhere (Saulnier et al., 2010; Barba et al., 2012). PCR products were qualitatively analyzed through electrophoresis on a 2% agarose gel.

Sequence-specific oligonucleotide primers were designed using Primer3 software (http://frodo.wi.mit.edu/primer3/); primer sequences are listed in Table 1.

**Table 1:** **Sequences of oligonucleotide primers used in rtPCR and qPCR**.

Gene name	Symbol	Sense^a^	5′-3′sequence
Glyceraldehyde-3-phosphate dehydrogenase	Gapdh	Fw	GCCCTCAATGACCACTTTGT
		Rv	TGCTGTAGCSGAACTCATTG
Chemokine (C-C motif) ligand 2	Ccl2	Fw	TAATACCCSGACCTGCTGT
		Rv	CATAACCCTTCACCCTCTTCA
Chemokine (C-C motif) ligand 5	Ccl5/Rantes	Fw	GTGGCCATGGGAAGTCTCTA
		Rv	TTGCCTTGAAAGATGTGCTG
Transforming growth factor beta	Tgfβ	Fw	GCCAAGGAGAGGAATTAGAGG
		Rv	SGGAGSGTGTTATCTTTGCT
Platelet-derived growth factor subunit A-like	Pdgfa	Fw	AGCATTGAGGAAGCCATTC
		Rv	CAGGAAGTTGGCAGASGTAG
Vascular endothelial growth factor A	Vegfa	Fw	CAAGATCSGCAGASGTGTAA
		Rv	CAASGSGAGTCTGTGTTTCT
Vascular endothelial growth factor receptor 1	Vegfr1	Fw	GSGSGAAACATCCTTCTATC
		Rv	CCAGGCCAAAATCACAAATC
Vascular endothelial growth factor receptor 2	Vegfr2	Fw	GCATGGAAGAGGATTCAGGA
		Rv	TCCTCSGTACAGGAAACAGG
Vascular endothelial growth factor receptor 3	Vegfr3	Fw	TTGAAGAATTCCCCATGACC
		Rv	TGATTATCCACAGGGGCTTT

### Survival of transplanted green fluorescent ASCs in the cochlea

After the final ABR test [see Auditory Function Evaluation (Auditory Brainstem Responses, ABR)], animals were deeply anesthetized (ketamine hydrochloride 25 mg/kg, xylazine 5 mg/kg, and acepromazine maleate 1.5 mg/kg body weight) and perfused transcardially with 0.9% NaCl (room temperature), followed by 4% paraformaldehyde at day 7 after surgery. The cochleae were quickly removed under a dissecting microscope (Zeiss Stemi DV4 Stereo Microscope, Jena, Germany) and processed for fluorescence and immunofluorescence analyses (described below). After 24 h of incubation with 4% paraformaldehyde in PBS at 4°C a pH 7.5, the cochleae were decalcified for 15 days (10% EDTA, changed daily), incubated for 48 h in cold 30% sucrose, and embedded in OCT. Frozen tissue samples were then cut into 16 μm thick sections using a Leica CM1850 cryostat (Leica Biosystems, Wetzlar, Germany). Five/ten neighboring randomly selected midmodiolus slices per cochlea were used to analyze the expression of transgenic GFP expression, as a reporter for ASC presence and survival; DAPI labeling was used to identify condensed cell nuclei. The analysis was performed using a fluorescence microscope equipped with a digital camera (Olympus BX63, Tokyo, Japan) and a confocal laser scanning system (TCS-SP2; Leica Microsystem, GmbH, Germany).

### Immunofluorescence

Immunofluorescence analysis was performed to evaluate the expression of the vascular endothelial growth factors A and C (VEGF-A and -C), the platelet-derived growth factor receptor (PDGFR), and the transforming growth factor β (TGFβ) in the *stria vascularis*, organ of Corti, and spiral ganglion. To this aim, tissue sections were incubated in a blocking solution containing 1% fatty acid-free bovine serum albumin (BSA), 0.5% Triton X-100, and 10% rabbit serum in PBS for 1 h at room temperature (Fetoni et al., 2010, 2011, 2013; Podda et al., 2013). The specimens were then incubated overnight at 4°C with a solution containing rabbit polyclonal anti-VEGF-A (diluted 1:100, Bioss Woburn, MA, USA), anti-VEGF-C (diluted 1:50, Santa Cruz Dallas, TX, USA), anti-PDGFR (diluted 1:500, Abcam Cambridge, UK), and anti-TGFβ1 (diluted 1:100, Antibodies, Atlanta, GA, USA) primary antibodies. All slides were then washed twice in PBS and incubated at room temperature for 2 h, light-protected, with labeled conjugated goat anti-rabbit secondary antibody (Alexa Fluor 633, IgG; Invitrogen, Carlsbad, CA, USA) diluted 1:400 in 0.1M PBS. After another wash in PBS, samples were double stained with DAPI (blue fluorescence, 1:500) for 20 min in the dark at room temperature. The slides were coverslipped with an antifade medium (ProLong Gold; Invitrogen). Immunolabeled specimens were analyzed using fluorescence microscopy and confocal imaging. Control experiments were performed by omitting the primary antibodies during processing of tissue randomly selected across experimental groups. Staining was absent in all the cochlear structures, indicating neither spontaneous fluorescence nor non-specificity of antibodies. All specimens were always processed together to limit variability related to antibody penetration, incubation time, post-sectioning age, and condition of tissue. Intensity of immunostaining was evaluated by three blinded independent observers who performed two measurements, as previously described (Picciotti et al., 2006). Briefly, the specimens were coded and the measurements were made without knowledge of animal data. A semiquantitative analysis of the intensity of the staining was performed at high magnification (100×), by using a visual numeric scale, ranging from 0 (negative staining) to 10 (most intense staining). Results were calculated as mean across animals, sections, and observers and classified as follows: <3: faint staining (+), between 3 and 7: moderate staining (+ +), and >7: intense staining (+ + +). To avoid possible biases caused by the selection of the fields to be examined, we selected the areas suitable for analysis on the basis of the integrity of the tissue.

### Statistical analysis

The results are presented as means ± SEM. Differences were assessed by using ANOVAs. Namely, ABR data were analyzed by means of three-way ANOVA (group × day × frequency). *Post hoc* comparisons were assessed with Tukey’s tests (StatSoft, Padua, Italy). Values of *p* < 0.05 were considered statistically significant. The statistical significance of the differences across the replicates in qPCR experiments was calculated using paired *t*-test on ΔΔ*Ct*, with a *p*-value cut-off of 0.05.

## Results

No signs of respiratory distress and weight loss, or mortality, were evident in all animals. No middle ear infection or vestibular dysfunction was observed during the first day after the surgical procedure. A complete healing of the surgical wound was achieved at day 5 after surgery. After the sacrifice, the *otic bullae* were examined by using the dissecting microscope and normal middle ear cavities were found in all animals, in particular no blood effusion or inflammation were found inside the cochlear duct in any animal, indicating that no major trauma was caused by the transplantation procedure itself.

### Functional evaluation

**Figure 2:**
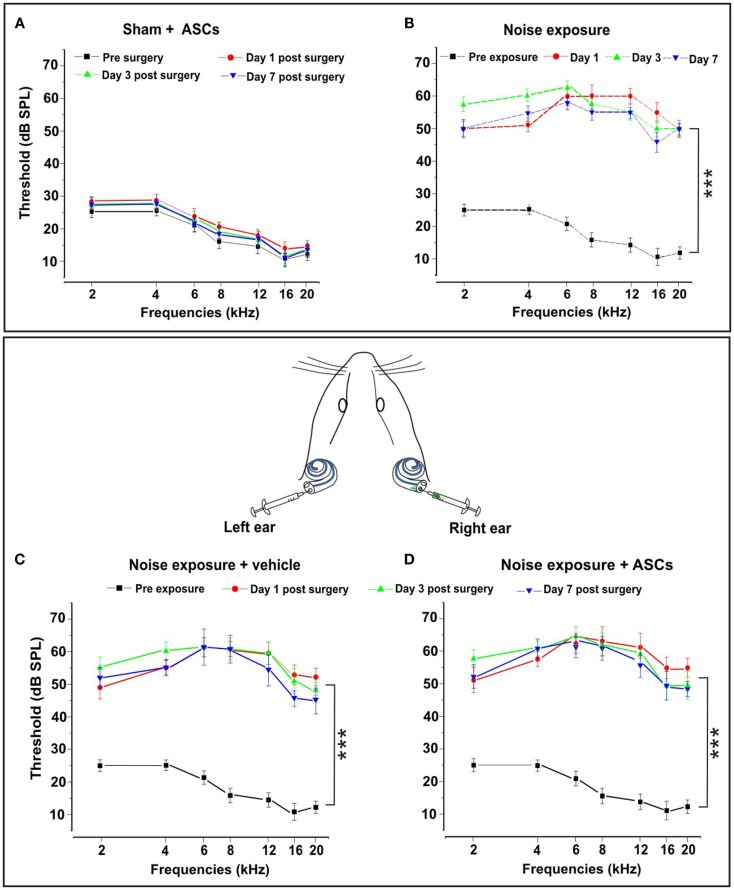
**Functional results**. The graphs in the upper panel show hearing threshold values in the Sham + ASCs and Noise-exposed groups. **(A)** ASCs implantation in unexposed animals does not affect significantly hearing threshold measured pre-surgery and 1, 3, and 7 days after surgery (*p* > 0.05). **(B)** Noise exposure causes a significant threshold elevation (*p* < 0.001) of about 40–45 dB in mid–high frequencies 1, 3, and 7 days after the end of noise exposure. The bottom panel shows the auditory mean threshold value measured in noise exposure + vehicle ear (left graph) and the noise-exposed + ASC implantation ear (right graph) before noise exposure and 1, 3, and 7 days after surgery. **(C)** Animals exposed to noise + vehicle show a threshold elevation of about 40–45 dB with no significant differences compared to noise-exposed animals. **(D)** ASCs implantation does not worsen the functional damage caused by noise.

To verify the reliability of our deafening procedure, and the effects of ASC graft on hearing, functional analyses were performed by ABR measurements. All data are expressed in terms of threshold and threshold shift, which represents the difference between the pre-noise and post-noise exposure value in each animal. No significant threshold shift was observed in control unexposed animals and in both right and left ears of sham controls injected with ASCs (Figure 2A) or vehicle (data not shown). The sham controls underwent surgery for ASC implantation without noise exposure, the threshold values remained stable in each time point as compared to pre-implantation. However, consistent with previous data, in the noise-exposed animals (Figure 2B) and those injected with vehicle (Figure 2C) after noise exposure, the greatest hearing loss occurred in the 6–12 kHz region, centered around the frequency of acoustic trauma (Fetoni et al., 2004, 2009, 2010). Namely, 24 h after the end of noise exposure, the threshold shift increased to about 40–45 dB for intermediate and high frequencies, respectively, and to 25–30 dB for the low frequencies. The threshold shift remained stable at day 3 after surgery. At day 7, there was a partial recovery of about 5–10 dB, with a greater attenuation in the high frequency range. The same pattern of threshold shifts was detected in the right ears of ASC-implanted noise-exposed animals (Figure 2D). In addition, no statistical differences were observed among ears of noise-exposed animals, left ears of vehicle injected noise-exposed animals, and right ears of ASC-implanted noise-exposed guinea pigs (compare Figures 2B–D). Taken together, these data indicated that the technical procedure used to perform cell inoculation did not cause neither any alteration of hearing or any additional hearing deficit, beyond that induced by the noise exposure.

### Differential gene expression in cochlear regions induced by the acoustic trauma

In order to assess the effect of noise exposure on cochlear tissues, with regard to the possible activation of pathways involved in trophism, regeneration, and cell migration, we have analyzed the expression of selected genes using qPCR in microdissected regions (organ of Corti, *stria vascularis*, spiral ganglion) of the cochlea of both noise-exposed and unexposed guinea pigs, prior to cell transplantation.

**Figure 3:**
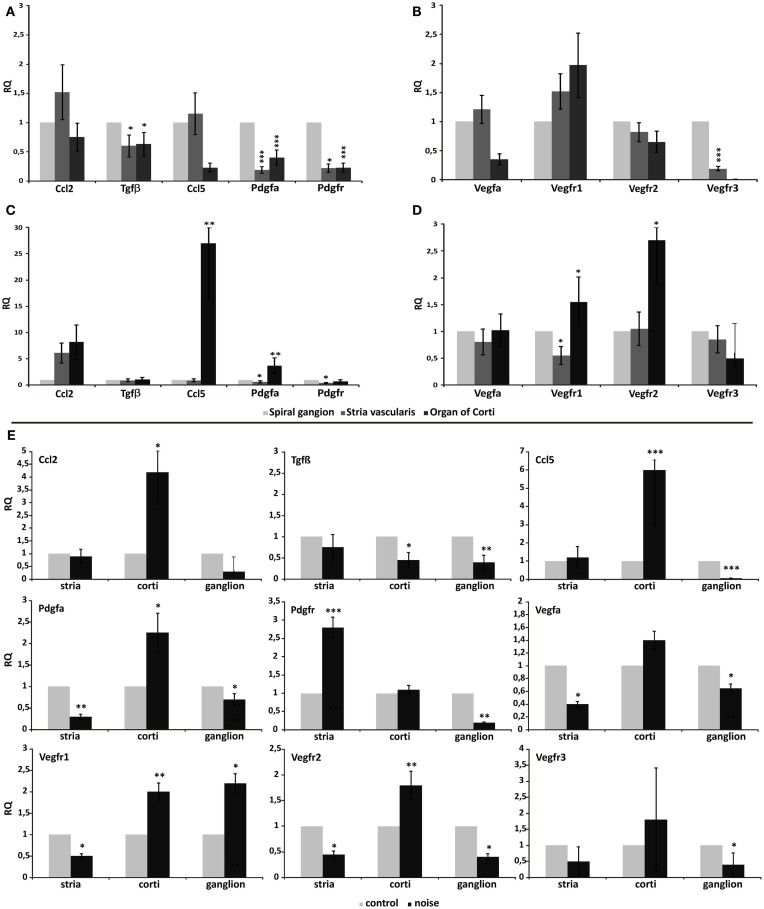
**Differential gene expression across different cochlear regions**. Graphs display the results obtained using quantitative real-time PCR to analyze the expression of genes in the three microdissected regions of the cochleae of control animals **(A–B)** and noise-exposed animals **(C–D)**. In **(E)**, real-time PCR data are shown from the paired analysis of control versus noise-exposed animals, in each tested region: *stria*, *stria vascularis*; corti, organ of Corti; ganglion, spiral ganglion. In each panel, relative quantity (RQ) refers to the mean levels of gene expression across replicates, calculated using the ΔΔ*Ct* method (see text for details).

First, we have analyzed comparatively the relative gene expression levels across the three cochlear regions, to explain tissue-specific profiles possibly involved in driving cell migration path, in control cochleae (Figures 3A,B), and in noise-exposed cochleae (Figures 3C,D). This revealed that the basal expression of Tgfβ, Ccl2, Ccl5/Rantes (Figure 3A), Vegf-A, and Vegfr2 (Figure 3B) genes did not vary significantly across the three microdissected regions, in control animals. Conversely, the expression of Pdgfa, Pdgfr (Figure 3A), and Vegfr3 (Figure 3B) was significantly reduced in the organ of Corti and in the *stria vascularis*, compared to the spiral ganglion. Finally, Vegfr1 expression was significantly higher in the organ of Corti, compared to the other regions (Figure 3B).

Upon noise exposure, the expression of Ccl5/Rantes, PdgfA (Figure 3C), and Vegfr2 (Figure 3D) significantly increased in the organ of Corti, compared to the *stria vascularis* and the spiral ganglion, upon noise exposure. The expression of Vegf-A did not vary significantly (Figure 3D). The organ of Corti expressed the highest level of Vegfr1 also in the noise-induced trauma (Figure 3D).

Thereafter, qPCR raw data were analyzed for each microdissected cochlear region, performing a pairwise comparison between the acoustic trauma (Noise group) and control groups (Control no-noise). This revealed that the expression of Ccl2 and Ccl5/Rantes was significantly increased only in the organ of Corti following acoustic damage, while remained unchanged between the experimental groups in the *stria vascularis* and spiral ganglion (Figure 3E). A change in Tgfβ expression was observed in both the organ of Corti and the spiral ganglion, being significantly downregulated in the Noise group compared to controls. PdgfA expression was significantly upregulated upon noise exposure only in the organ of Corti. Conversely, the expression of PdgfA in the *stria vascularis* and spiral ganglion was reduced in the acoustic trauma group (Figure 3E). Pdgfr expression was increased in noise-deafened animals compared to controls, only in the *stria vascularis* (Figure 3E). The analysis of Vegf-A and its receptors revealed that Vegf-A expression was reduced upon noise-induced injury compared to controls, in both the *stria vascularis* and the spiral ganglion. The expression of Vegfr1 was significantly increased in the organ of Corti and ganglion, while reduced in the *stria*, of noise-deafened animals compared to controls. Vegfr2 expression was increased only in the organ of Corti, while reduced in the other compartments, in noise versus control animals. Finally, the expression of the Vegfr3 was downregulated in the spiral ganglion of noise-deafened animals compared to controls (Figure 3E).

The expression of the growth factors and corresponding receptors was also analyzed qualitatively in ASCs using rtPCR, in order to assess their possible role in driving *in vivo* cell migration. The analysis showed that ASCs expressed Tgfβ, Vegf-A, PdgfA, and Pdgfr (see Figure 6O).

### Detection of ASCs in the cochlea

**Figure 4:**
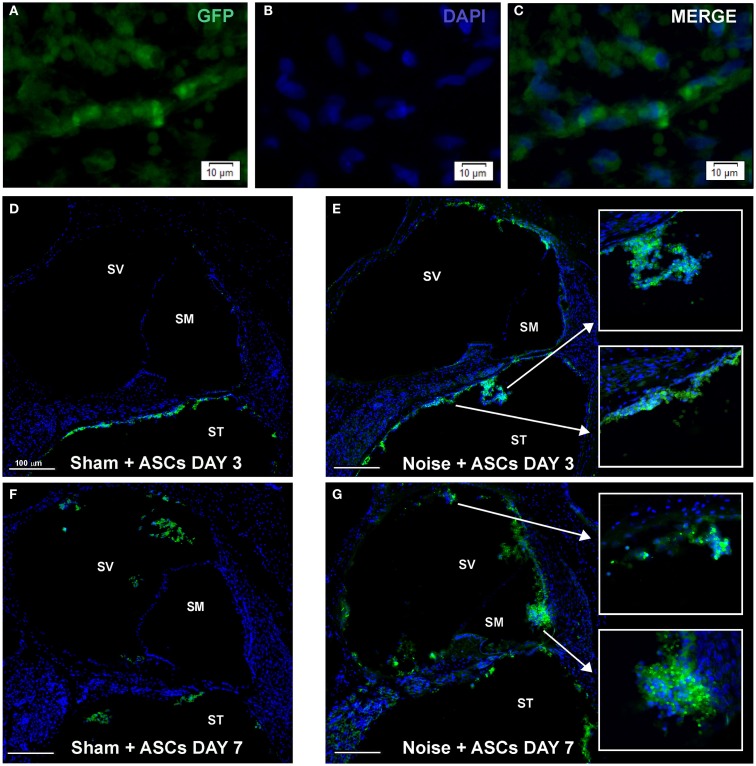
**Morphological results**. The figure shows the results obtained upon intracochlear inoculation of the GFP-expressing ASCs. **(A–C)** shows representative images of ASC aggregates floating in the perilymphatic liquid. **(D–G)** shows the location of GFP-positive cells in Sham + ASCs and Noise-exposed specimens 3 and 7 days after surgery (the cochlear mid-modiolar sections are stained whit DAPI). At day 3 ASCs are mainly detected neighboring the basilar membrane and the Rosenthal canal in the *scala tympani* (ST) both in Sham + ASCs **(D)** and in Noise + ASCs specimens [**(E)** magnification indicated by arrows]. At day 7, few GFP-positive cells are detected in ST and in *scala vestibuli* (SV) in Sham + ASCs section **(F)**, while in Noise + ASCs specimen **(G)** ASCs migrate into the *scala media* (SM) close to the *stria vascularis* and spiral ligament (see high magnification).

**Figure 5:**
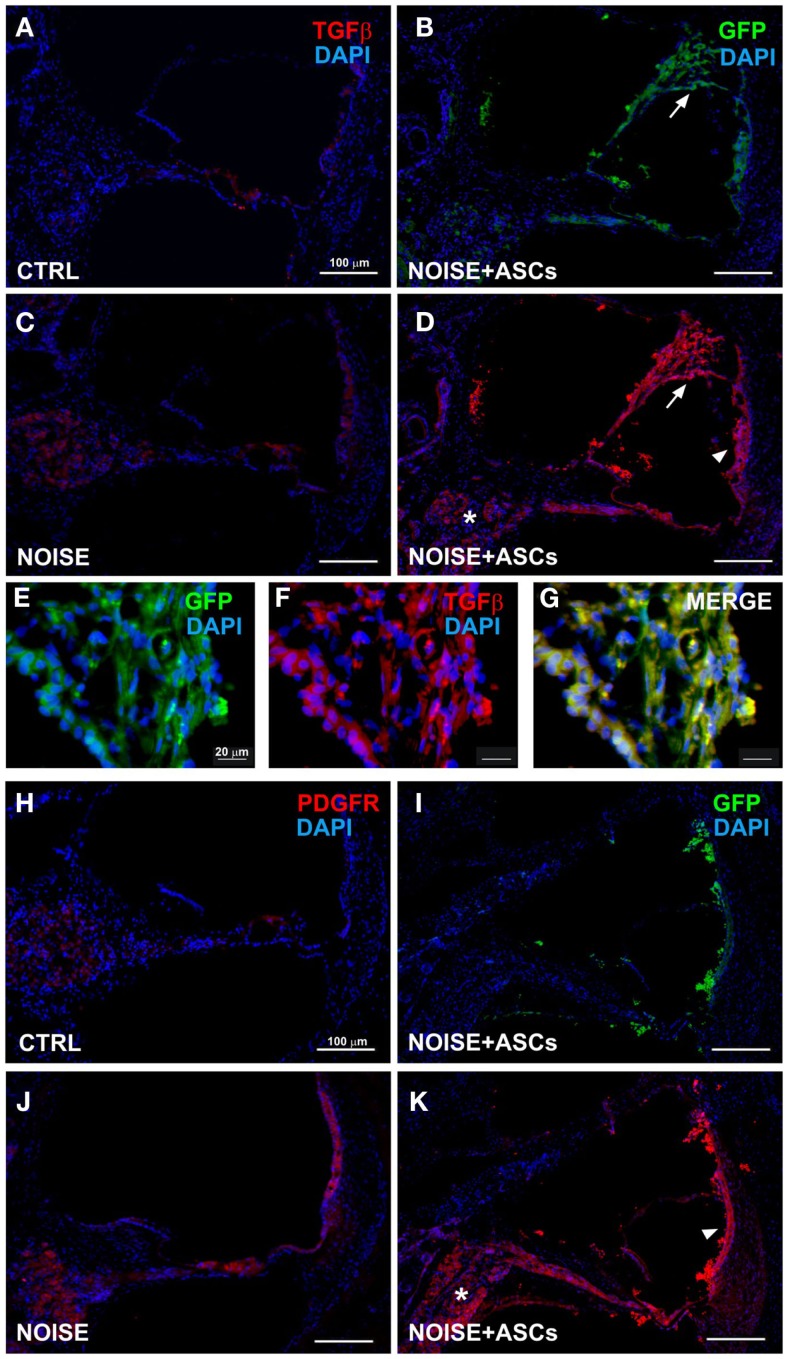
**TGFβ and PDGFR expression in the cochlea**. Representative images from confocal microscopy analysis of cochlear cryosections collected at day 7 after surgery. TGFβ (red) and DAPI nuclear staining (blue) in **(A)** control unexposed cochleae; **(B)** GFP-positive cells are located close to the Reissner’s membrane (arrow) in the *scala media* (the faint green fluorescence shown in cochlear structures was observed in control negative sections due to a slight spontaneous fluorescence, data not shown); **(C)** in the noise + vehicle group (TGFβ fluorescence observed in the spiral ganglion, organ of Corti and *stria vascularis*); **(D)** after ASC implantation, red fluorescence is observed in the *scala media* and in implanted ASCs near the lateral wall (*stria vascularis*/arrow head and spiral ligament), in the spiral ganglion (asterisk), and Reissner’s membrane (arrow), as shown at higher magnification **(E–G)**, where ASCs attached to the Reissner’s membrane are shown. In lower panels, PDGFR staining is shown in unexposed **(H)** and noise-exposed cochleae [**(J)** red fluorescence in the organ of Corti, spiral ganglion, and *stria vascularis*]; **(I)** green fluorescent ASCs in the noise-exposed cochlea; **(K)** PDGFR increased fluorescence in noise + ASCs group in the *stria vascularis* (arrow head) and in the spiral ganglion (asterisk). TGFβ, transforming growth factor β; PDGFR, platelet-derived growth factor receptor.

**Figure 6:**
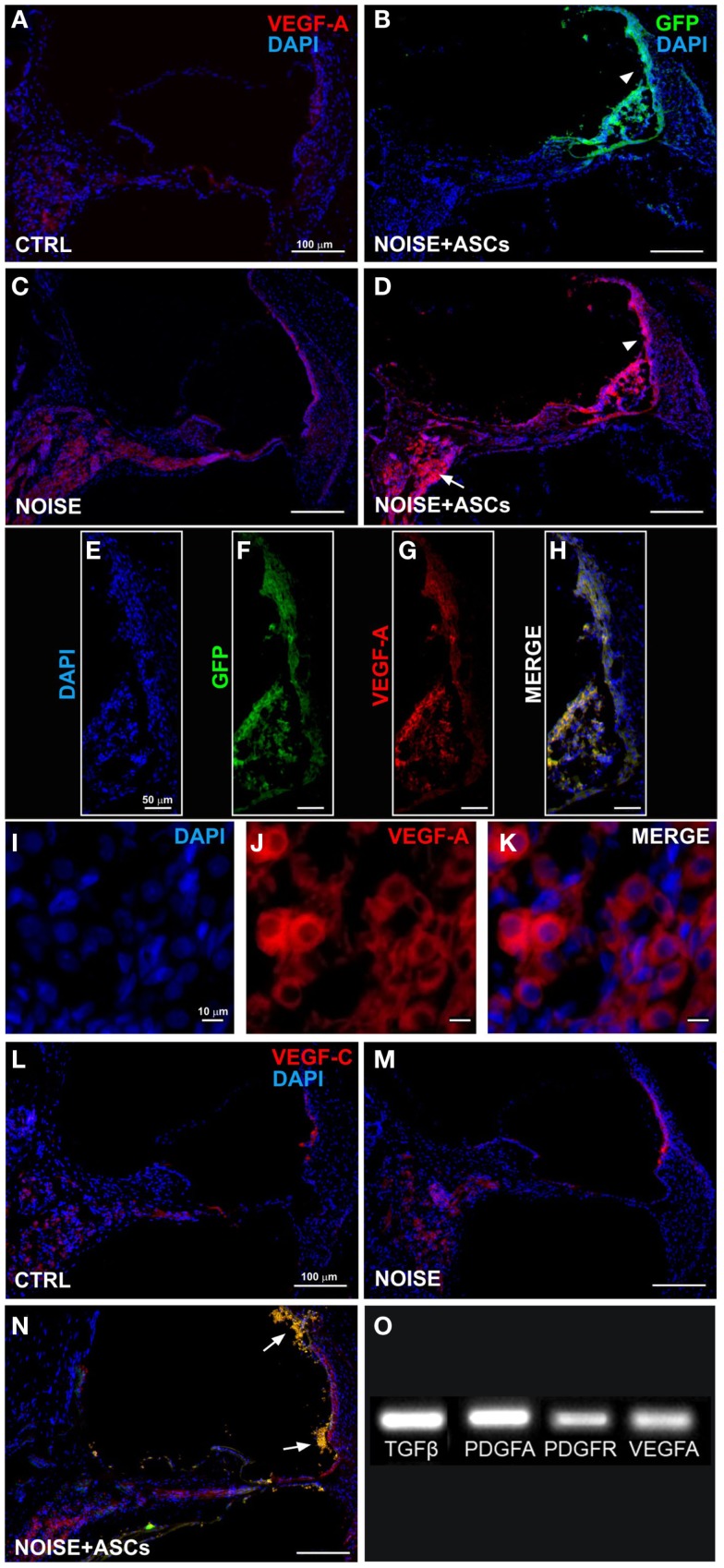
**VEGFs expression in the cochlea**. Representative images from confocal microscopy of cochlear cryosections collected at day 7 after surgery: **(A)** VEGF-A staining (red fluorescence) in control unexposed cochlea; **(B)** green fluorescent cells in the scala media (arrow head); **(C)** VEGF-A expression in noise-exposed; **(D)** increased VEGF-A expression in the *scala media* and in implanted ASCs near the *stria vascularis* (arrow head) and in the spiral ganglion (arrow); **(E–H)** higher magnification of the *stria vascularis*; and **(I–K)** of the spiral ganglion; **(L–N)** VEGF-C and GFP fluorescence in cochlear sections; ASCs close to the lateral wall (arrows). Panel **(O)** shows electrophoretic bands of cDNA migration, from rtPCR analysis performed in cultured ASCs. VEGF-A, vascular endothelial growth factor A; VEGF-C, vascular endothelial growth factor C.

Morphological analysis of cochlear sections revealed no signs of relevant traumatic damage to the basilar membrane, the organ of Corti, and the Reissner’s membrane, attributable to the surgical procedure, in any of the tested animals. ASCs were clearly distinguished from the endogenous cells based on their transgenic GFP expression, which co-localized with DAPI-stained nuclei (Figures 4A–C), and tended to aggregate close to each other, as observed in cochlear specimens 3 and 7 days after implantation (Figures 4D–G) and they were thus recognized as exogenous cells into the labyrinthine fluid compartments. Namely, at day 3, in the Sham + ASCs specimens, the transplanted cells were found as a cluster of green fluorescent ASCs in the perilymphatic spaces mainly attached to the walls of the *scala tympani* (Figure 4D); however, in the noise-exposed + ASCs ears, some ACSs migrated to the *scala vestibuli* (Figure 4E); few cells were also found attached to the basilar membrane in the *scala tympani* and in the proximity of Rosenthal canal (Figures 4D,E). At day 7, in the Sham + ASCs ears, the green fluorescent cells in the perilymphatic spaces of the *scala tympani* decreased, while they increased in the *scala vestibuli* (Figure 4F). Interestingly, in the noise-exposed + ASCs ears, the fluorescence was also found in the *scala media* close to the tectorial membrane and the *stria vascularis* indicating that ASCs migrated in the *scala vestibuli* and in the *scala media* (Figure 4G). Some ASCs were detected nearby the Reissner’s membrane (see also Figures 5 and 6).

### Growth factor expression in the cochlea upon ASC transplantation

Immunofluorescence was used on cochlear sections to detect and localize the expression of TGFβ, PDGFR, VEGF-A, and VEGF-C at day 7 after surgery. Implanted ASCs showed an intense positivity for VEGF A and C. A faint TGFβ expression was observed in control (unexposed, Figure 5A) and slightly increased in the Noise + vehicle ears (Figure 5C). No differences were detected either between control and sham ears or between noise-exposed and Noise + vehicle ears (data not shown). A marked expression of TGFβ was observed in the ASC-treated noise-exposed right ears, labeling both the implanted ASCs and the surrounding cochlear tissues (Figure 5D). Namely, the ASCs, showing a strong TGFβ positivity, were attached to the Reissner’s membrane and others localized in clusters in the *scala vestibuli* and *media* (Figure 5B). An increased expression of TGFβ was detected on the right ASC-implanted ears compared to Noise + vehicle left ears in the *stria vascularis*, organ of Corti, spiral ganglion, and fibers (Figures 5E–G). PDGFR levels increased in the Noise + vehicle ears (Figure 5J) as compared to controls (Figure 5H), in particular in the *stria vascularis*, the organ of Corti, and spiral ganglion. Namely, after ASC implantation, a marked increase of PDGFR fluorescence was observed (Figure 5K) as compared to the contralateral Noise + vehicle ears, mainly in the *stria vascularis* and spiral ganglion (Figure 5J). Furthermore, implanted ASCs in the *scala media* displayed an intense PDGFR expression (Figure 5K). No differences were detected either between control and sham ears or between noise-exposed and Noise + vehicle ears (data not shown). As shown in Figure 6, the expression of two members of the VEGF family, VEGF-A and VEGF-C, was analyzed in cochlear specimens using immunofluorescence. VEGF-A levels overall increased in the noise-damaged cochlea (Noise + vehicle, Figure 6C) compared to controls (Figure 6A), in particular in the *stria vascularis*, the organ of Corti, and spiral ganglion, confirming previous data (Picciotti et al., 2006). Interestingly, upon ASC inoculation, both cells and cochlear tissues showed an intense expression of VEGF A, mainly in the *stria vascularis* (Figures 6E–H) and spiral ganglion (Figures 6I–K). The expression of VEGF-C was observed in the same regions (Figures 6L–N). No differences were detected either between control and sham ears or between noise-exposed and noise + vehicle ears (data not shown).

Accordingly, rtPCR data indicated that ASCs expressed basal levels of Tgfβ, Pdgfa, Pdgfr, and Vegfa genes *in vitro*, prior to their implantation (Figure 6O).

## Discussion

Adipose-derived stem cells are being considered the most suitable tool in a wide range of regenerative medicine applications, though few studies investigated their use in the field of SNHL, so far. Rather than being able to transdifferentiate and drive a true regeneration of the damaged neuroepithelium, ASCs could be able to sustain tissue trophism and increase endogenous repair (Lattanzi et al., 2011). Due to their strong immunomodulatory properties, these cells have been indeed proved to be effective in systemic administration, for treating experimental autoimmune hearing loss, by improving hearing function, and protecting hair cells (Zhou et al., 2011).

In this study, we have demonstrated that direct *in vivo* inoculation of ASCs through the round window into the guinea pig cochlea, does not induce any sign of distress or structural damage to the cochlea, and does not affect auditory function. In addition, we have originally shown that ASCs survive upon intracochlear inoculation, and migrate from the perilymphatic to the endolymphatic compartment.

In particular, transplanted cells were found in the *scala tympani*, *scala vestibuli*, and *scala media*, while some had traveled from their implantation site in the basal turn to the apical turn. This movement could be attributable to active cell migration or fluid dynamics. ASCs were observed close to the noise-injured organ of Corti and the *stria vascularis*, although integration of cells into endogenous tissue was not observed.

The structural separation between the endolymphatic and perilymphatic compartments is represented by the reticular lamina, which provides a tight junction barrier restricting the interchange of materials between these regions (Pickles, 2001). It may be speculated that the ASC movement from the perilymphatic to the endolymphatic compartment could be due to migration across the reticular lamina, given that, following noise-related injury, the tight junctions are known to rearrange (Raphael and Altschuler, 1991; Hildebrand et al., 2005). Indeed, it is thus conceivable that during the rearrangement of these tight junctions, between the formation of phalangeal scars and the restoration of the reticular lamina, transplanted ASCs would be able to cross the reticular lamina into the organ of Corti.

In addition, active migration of MSCs has been widely described, being mediated by the TGFβ axis. Our data could not demonstrate cell migration, but indicated that cultured ASCs express, prior to *in vivo* inoculation, the Tgfβ gene, among the array of growth factors that may contribute to their trophic features.

Our gene expression data also suggested that the multifunctional cytokine TGFβ appeared to be stably expressed in recipient cochlear tissues, prior to ASC inoculation, although without any clear increase induced by noise exposure. It has been widely demonstrated that the TGFβ protein is sequestered in a latent form in extracellular matrix, from where it is released as active molecule in response to perturbations induced by mechanical stress, wound repair, tissue injury, and inflammation (Munger et al., 1997; Kanzaki et al., 1998; Annes et al., 2003). Upon the injury-induced activation, active TGFβ controls the mobilization and recruitment of MSCs to participate in tissue repair and remodeling (Wan et al., 2012). Once MSCs have reached the injured site, they are able to promote angiogenesis and tissue regeneration, through inducing the cascade expression of chemokines, including the chemokine (C-C motif) ligand 2 (CCL2), that amplifies the signal for cell migration (Wan et al., 2012). Interestingly, our data indicated that Ccl2 gene was strongly upregulated in the damaged organ of Corti, upon noise stimulation. CCL2, also referred to as monocyte chemotactic protein 1 (MCP1), is an inflammatory cytokine, recently involved in homing and migration of MSCs into organ/tissue-specific locations (Belema-Bedada et al., 2008; Xu et al., 2010). Also, the chemokine (C-C motif) ligand 5 (CCL5) was upregulated in the damaged organ of Corti, following noise exposure. CCL5, also known as RANTES (regulated on activation, normal T cell expressed and secreted) is indeed another chemotactic cytokine, able to mediate adipose-derived stem cell migration (Kroeze et al., 2009). In particular, CCL5-related signaling is also involved in regulating ASC stemness and multilineage potential, through the modulation of the stemness-related genes, SOX2, OCT4, and NANOG, expression (Kauts et al., 2013). Confocal microscopy indicated an increase in TGFβ expression in noise-exposed animals receiving ASC inoculation.

Based on this background, we may hypothesize that in the present model, ASCs could be able migrate toward the injured organ of Corti, chemoattracted by CCL2, and there amplify the TGFβ-dependent chemokine cascade. Though a detailed study aimed at tracking the *in vivo* cell migration path into the cochlear compartment would be needed to confirm this assumption.

In this study, we have demonstrated that cultured ASCs also expressed trophic growth factors, involved in several pathways that play significant roles in tissue regeneration. In particular, ASCs express *in vitro* the genes encoding both the PDGF and the corresponding receptor PDGFR. Confocal microscopy showed that noise exposure apparently induced an increased immunofluorescence of PDGFR in the organ of Corti, *stria vascularis*, and spiral ganglion. Gene expression analysis, performed in recipient cochlear tissues before cell inoculation, confirmed an increase of PDGFR expression induced upon noise exposure only in the *stria vascularis*. Interestingly, a marked increase of PDGFR fluorescence was observed in noise-injured cochleae upon ASC inoculation. PDGF has an active role in enhancing migration, proliferative responses, and extracellular matrix synthesis mesenchymal cells (Ozaki et al., 2007; Donovan et al., 2013). Specifically, the angiogenic potential of ASCs relies prominently on PDGF that is released upon angiogenic induction (Dhar et al., 2010), induces their differentiation (Ball et al., 2010; Lopatina et al., 2014), and increases the release of pro-angiogenic microvesicles (Pallua et al., 2014). Interestingly, previous studies indicated that integrity of PDGF signaling is required for the proliferation of developing cochlear hair cells (Lee et al., 2004), for the trophism of the vascular and mesenchymal compartment in the neonatal mouse inner ear and, indirectly, for the survival of the sensory epithelium (Hayashi et al., 2008).

We have previously demonstrated that VEGF has a leading role in regulating the vascular network of the inner ear, after acoustic trauma and during auditory recovery (Picciotti et al., 2006; Fetoni et al., 2009). In particular, in our previous study, VEGF protein levels showed an overall increase in the cochlea, following noise exposure. In the present study, gene expression data indicated that (1) ASCs expressed VEGF-A *in vitro* prior to implantation and (2) noise exposure induced an increased expression of the VEGF receptors specifically in the injured organ of Corti, suggesting an increased sensitization of this region to the action of the ligand VEGF. The strong trophic potential of ASCs is believed to reside mainly in their angiogenic properties (Song et al., 2010); these cells are indeed able to promote wound healing through angiogenesis and vasculogenesis (Nie et al., 2011) and are known to secrete a plethora of growth factors, including VEGF, that leads this process (Salgado et al., 2010).

Overall, the experimental design exploited in this study was intended to obtain preliminary evidences on the feasibility of an ASC-based transplantation procedure into the cochlea. Our data demonstrated that the proposed approach is feasible in terms of (a) safety of implantation in preclinical setting and (b) cell survival in the perilymphatic and endolymphatic fluids. Further studies are compulsory to demonstrate the possibility of hearing improvement obtained through an ASC-based approach and to quantify possible long term effects.

Relevant translational consequences may plausibly derive from the capability of ASCs to express PDGF and VEGF, considering the need for a therapeutic approach to cochlear regeneration, enabling a sustained local delivery of growth factors, which overcome their short half-life (Bowen-Pope et al., 1984). In this context, the transplantation of cells acting as a viable reservoir for the endogenous synthesis and secretion of trophic molecules, may increase the local availability of growth factors, without the need of repeated exogenous administration.

In a translational perspective, the feasibility of local ASCs delivery could pave the way to future innovative biological strategies, also in combination with conventional therapies, such as cochlear implantation, to enhance the endogenous reparative processes in the early stage of injury.

## Author Contributions

All authors provided substantial contributions to the work. In particular, Anna Rita Fetoni and Wanda Lattanzi conceived and designed the work, interpreted the results, drafted the manuscript, and revised it critically; Sara Letizia Maria Eramo, Chiara Moriconi, Marta Barba, Fabiola Paciello, and Rolando Rolesi collaborated in data acquisition and analysis, and drafting of the manuscript; Diana Troiani collaborated in data interpretation, manuscript drafting, and critical revision; Fabrizio Michetti, Diana Troiani, and Gaetano Paludetti provided senior consult in data interpretation and critical revision of the manuscript. Finally, all authors approved the final version of the manuscript to be published and agreed to be accountable for all aspects of the work.

## Conflict of Interest Statement

The authors declare that the research was conducted in the absence of any commercial or financial relationships that could be construed as a potential conflict of interest.

## Supplementary Material

The Supplementary Material for this article can be found online at http://www.frontiersin.org/Journal/10.3389/fncel.2014.00334/abstract
