# Supplementary material for: Grafting and Early Expression of Growth Factors from Adipose-Derived Stem Cells Transplanted into the Cochlea, in a Guinea Pig Model of Acoustic Trauma
Source: Front Cell Neurosci. 2014 Oct 20;8:334. doi: 10.3389/fncel.2014.00334 (PMC4202717; doi:10.3389/fncel.2014.00334)
Supplement: Supplementary file 1 [file Data_Sheet_1.DOCX]

Semi-quantitative detection of growth factor/receptor expression in the different cochlear regions, in “noise”-over-“noise+ASCs” groups. Scores were assigned by independent observers, as specified in the manuscript text.

| [**Cochlear**](javascript:void(0);) **region TGFB expression** | **Observer 1** | **Observer 2** | **Observer 3** |
| --- | --- | --- | --- |
| Organ of Corti* | ++ | ++ | ++ |
| [Spiral ganglion](javascript:void(0);) | ++ | ++ | ++ |
| Stria vascularis | +++ | +++ | +++ |
|  | | | |
| [**Cochlear**](javascript:void(0);) **region PDGFR expression** | **Observer 1** | **Observer 2** | **Observer 3** |
| Organ of Corti* | ++ | ++ | ++ |
| [Spiral ganglion](javascript:void(0);) | ++ | ++ | ++ |
| Stria vascularis | +++ | +++ | +++ |
|  | | | |
| [**Cochlear**](javascript:void(0);) **region VEGFA expression** | **Observer 1** | **Observer 2** | **Observer 3** |
| Organ of Corti* | + | + | + |
| [Spiral ganglion](javascript:void(0);) | +++ | +++ | +++ |
| Stria vascularis | +++ | +++ | +++ |
|  |  |  |  |
| [**Cochlear**](javascript:void(0);) **region VEGFC expression** | **Observer 1** | **Observer 2** | **Observer 3** |
| Organ of Corti* | + | + | + |
| [Spiral ganglion](javascript:void(0);) | + | + | + |
| Stria vascularis | ++ | ++ | ++ |

*: neuroepithelium area
