# Supplementary figures and images for: Grafting and Early Expression of Growth Factors from Adipose-Derived Stem Cells Transplanted into the Cochlea, in a Guinea Pig Model of Acoustic Trauma
Source: Front Cell Neurosci. 2014 Oct 20;8:334. doi: 10.3389/fncel.2014.00334 (PMC4202717; doi:10.3389/fncel.2014.00334)

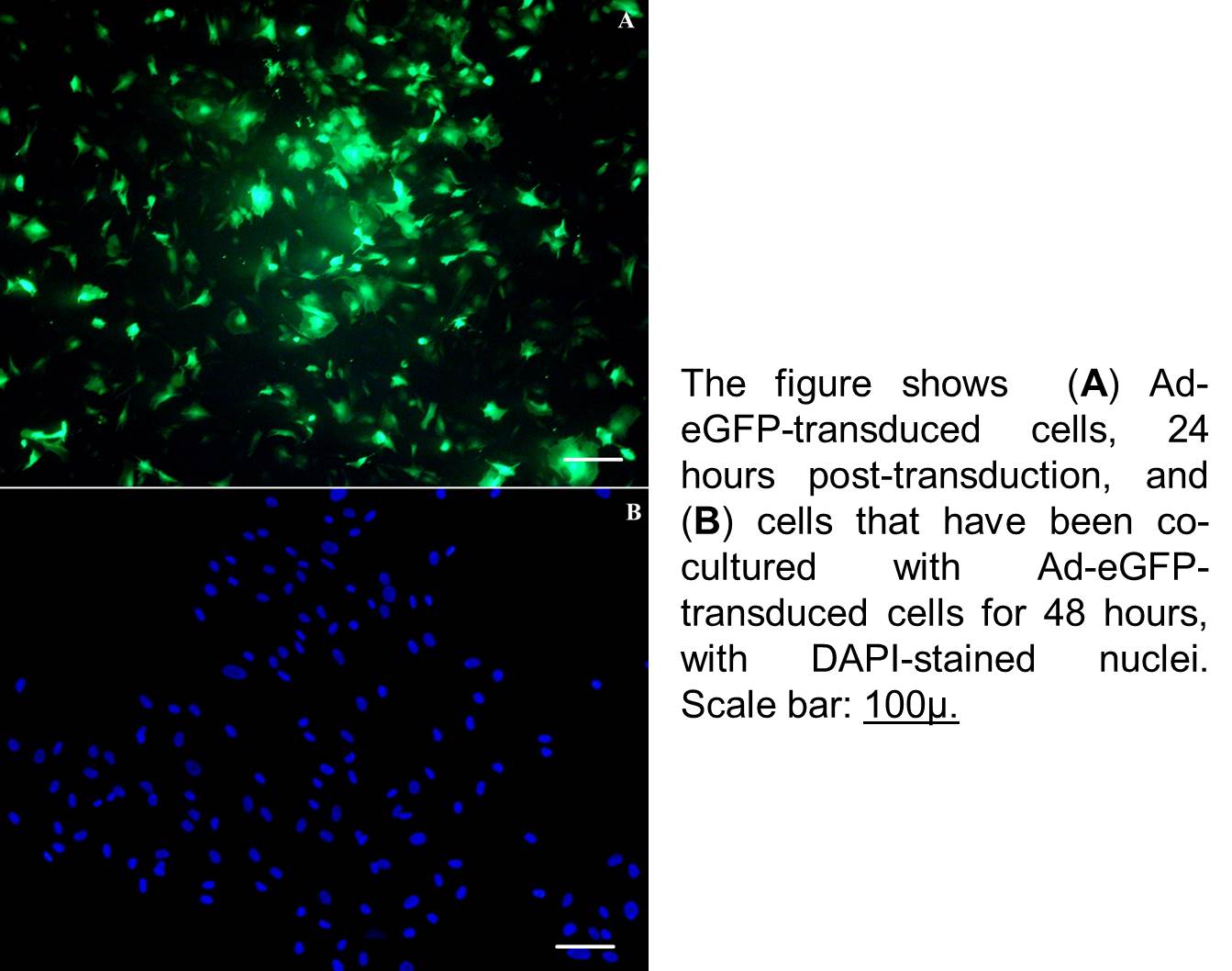

Supplement: Supplementary file 2 [file Image_1.JPEG]
